# Strategies to Improve the Quality of Goat Yogurt: Whey Protein Supplementation and Milk Pre-Treatment with High Shear Dispersion Assisted by Ultrasound

**DOI:** 10.3390/foods13101558

**Published:** 2024-05-16

**Authors:** Lorena Soares Xavier, Flaviana Coelho Pacheco, Gabriela Aparecida Nalon, Jeferson Silva Cunha, Fabio Ribeiro dos Santos, Ana Flávia Coelho Pacheco, Alline Artigiani Lima Tribst, Bruno Ricardo de Castro Leite Júnior

**Affiliations:** 1 Department of Food Technology (DTA), Federal University of Viçosa (UFV), Viçosa 36570-900, MG, Brazil; lorena.xavier@ufv.br (L.S.X.); flaviana.pacheco@ufv.br (F.C.P.); gabriela.nalon@ufv.br (G.A.N.); jeferson.cunha@ufv.br (J.S.C.); fabio.r.santos@ufv.br (F.R.d.S.); 2 Instituto de Laticínios Cândido Tostes, Empresa Agropecuária de Minas Gerais (EPAMIG), Tenente Luiz de Freitas, 116, Juiz de Fora 36045-560, MG, Brazil; ana.pacheco@epamig.br; 3 Núcleo de Estudos e Pesquisas em Alimentação (NEPA), Universidade Estadual de Campinas (UNICAMP), Campinas 13083-852, SP, Brazil; tribst@unicamp.br

**Keywords:** emerging technologies, non-bovine milk, fermentation, protein fortification, yogurt stability, rheology

## Abstract

This work investigated the fermentation kinetics and characteristics of goat yogurt supplemented with bovine whey protein isolate (WPI) (0%, 2.5% and 5.0%) subjected to high shear dispersion (HSD) assisted by ultrasound (US). Protein supplementation and the physical processes increased the electronegativity of the zeta potential (≤60%), whereas particle size reduction was observed only with physical processes (≤42%). The addition of 2.5% WPI reduced yogurt fermentation time by 30 min. After 24 h of storage at 7 °C, lactic acid bacteria counts did not differ between samples (≥8 log CFU/mL), and the supplementation was sufficient to increase the apparent viscosity (≤5.65 times) and water-holding capacity (WHC) of the yogurt (≤35% increase). However, supplementation combined with physical processes promoted greater improvements in these parameters (6.41 times in apparent viscosity and 48% in WHC) (*p* < 0.05), as confirmed by the denser and better-organized protein clusters observed in microscopic evaluation. Thus, both approaches proved to be promising alternatives to improve goat yogurt quality. Therefore, the decision to adopt these strategies, either independently or in combination, should consider cost implications, the product quality, and market demand.

## 1. Introduction

Goat milk production is growing worldwide, and it is expected to have a 53% increase by 2030 [1,2]. Compared to cow milk, goat milk has smaller fat globules and lower αs1-casein content, which improves milk digestibility and reduces its allergenicity, respectively, compared to cow milk [3,4]. Additionally, this milk contains health-promoting compounds such as conjugated linoleic acids, oligosaccharides, and bioactive peptides [5].

Despite the benefits regarding lower allergenicity, the low content of αs1-casein in goat milk negatively affects yogurt consistency and water-holding capacity [6,7]. In this scenario, different strategies have been used to improve the textural properties of goat yogurt, including the incorporation of thickeners and fibers [5,8,9,10], the application of protein-modifying enzymes, such as microbial transglutaminases [11], and treatment with physical processes able to alter the structure and interactions of the protein matrix and fat globules [4].

Ultrasound (US) and high shear dispersion (HSD) are potential emerging technologies to be used in goat milk to enhance the rheological aspects of the yogurt produced. US is an operationally simple, relatively low-cost, non-toxic, and environmentally friendly technology [12]. Sonication can induce partial protein denaturation, as well as reduce the size of cow milk fat globules, thereby reducing yogurt fermentation time and improving water retention capacity, gel viscosity, and the reduction of syneresis [12,13,14]. However, goat milk pre-treatment with US to improve yogurt quality remains underexplored, with works restricted to probe ultrasonic application [15,16], which faces challenges in scalability and durability due to probe tip wear [17]. Moreover, scaling up probe-based ultrasonic systems is problematic, as intensity diminishes exponentially away from the probe, compromising process uniformity [18]. Therefore, this study utilized bath ultrasonication for its cost-effectiveness, scalability, and process uniformity [19]. The high shear dispersion (HSD), also known as rotor–stator mixer or high shear homogenizer, produces high shear rates due to the centrifugal forces given by the equipment rotation [20]. These high velocity gradients can reduce the fat globule size of milk and can have a multifaceted impact on goat milk proteins, influencing their structure, solubility, and interactions within the matrix [21], potentially improving the gel quality. Therefore, alongside bath ultrasonication, HSD was considered in this study for its ability to offer cost-effective, scalable, and uniform treatment, complementing the benefits of ultrasound treatment [19].

Protein supplementation is another strategy to increase yogurt consistency. The use of whey proteins has stood out for this propose, as its incorporation promotes textural improvements and provides health benefits due to its functional properties [22,23,24].

Although these strategies are listed in the literature, no previous study has investigated the outcome magnitude of each strategy alone or together to improve the overall quality of goat yogurt. Therefore, considering the importance of this subject from an industrial and scientific perspective, this work evaluated the effect of goat milk supplementation with whey protein and/or milk pre-treatment with HSD assisted by US, focusing on the fermentation profile and physicochemical, rheological, and microstructural characteristics of the goat yogurt.

## 2. Materials and Methods

### 2.1. Goat Milk, Whey Supplementation and Cultures

Raw goat milk was acquired from the goat-farming sector of the University of Viçosa. For supplementation, a commercial bovine whey protein isolate (WPI) (NewNutrition, Ribeirão Preto, Brazil) with 90% protein was used. The commercial yogurt culture SLB 95 (Diagrama, Santa Fe, Argentina) with *Streptococcus salivarius* subsp. *thermophilus* and *Lactobacillus delbrueckii* subsp. *bulgaricus* was used for milk fermentation.

### 2.2. Milk Pre-Treatment by Ultrasound-Assisted High Shear Dispersion (US-HSD)

One-liter portions of goat milk were separated and WPI was added at different concentrations (0%, 2.5% and 5%) (*w*/*v*). At room temperature, samples were stirred using a magnetic stirrer (Fisatom, model 752A/6, São Paulo, Brazil) at 700 rpm for 5 min. Subsequently, the mixtures were subjected to high shear dispersion pre-treatment (Ultra-Turrax^®^, model T-18 Basic, Ika, Staufen, Germany) at 15,000 rpm, assisted by ultrasound for 10 min at 40 °C. The ultrasonic bath (Unique, model USC 2800 A, São Paulo, Brazil) used to couple the high shear disperser had temperature control, volumetric capacity of 9.5 L, frequency of 25 kHz and volumetric power of 38 W/L, verified using the calorimetric method [25]. For comparative purposes, supplemented samples (0%, 2.5%, and 5% WPI) not subjected to physical processes were placed in a thermostatic bath at 40 °C for 10 min. The sample not supplemented (0% WPI) and not subjected to physical processes was defined as the control.

### 2.3. Mean Particle Size and Zeta Potential of Goat Milk after Pre-Treatment by US-Assisted HSD

The mean particle size (MPS) and zeta potential (ζ) of the samples were determined after the application of the physical processes, following the procedures reported by Soares et al. [26], but modifying the dilution to 1:100, and using water as the diluent. The analyses were carried out at 25.0 ± 0.1 °C on the Zetasizer Nano ZS equipment (Malvern Instruments Ltd., Worcestershire, UK).

### 2.4. Goat Yogurt Fermentation

After pre-treatments, the samples were pasteurized (80 °C/30 min) [27], followed by cooling to 42 °C for the inoculation of the yogurt culture at 10^6^ CFU/mL. After yogurt culture inoculation, the samples were fermented at 42 °C and the pH of the samples was measured at 30 min intervals until the samples reached a pH of 4.6. The pH values were adjusted using the Gompertz equation (Equation (1)) [28] to obtain the lag phase (λ) and the maximum rate of the pH decrease (μ) as follows

(1) $$pH={pH}_{0}+\left({pH}_{\infty }-{pH}_{0}\right)exp\{-exp[\frac{µe}{\left({pH}_{\infty }-{pH}_{0}\right)}\left(\lambda -t\right)+1]\}$$

where pH_0_ = pH at the beginning of fermentation, pH∞ = pH at the end of fermentation, µ = maximum rate of pH decrease (h^−1^), λ = lag phase (h), and e (Euler number) = mathematical constant, approximately equal to 2.71828, and t = time (h). The parameters λ and μ from Equation (1) were obtained through non-linear regressions using CurveExpert Professional software (version 2.6.5, Hyams Development, Chattanooga, TN, USA) at a 95% confidence level (*p* < 0.05).

The yogurts were stored at 7 °C for 24 h and then stirred using a metal spatula (30 times clockwise; 30 times counterclockwise) to homogenization [27]. Then, the viability of lactic acid bacteria and physicochemical, rheological, microstructural analyses were determined.

### 2.5. Physicochemical Analyzes and LAB Viability

The pH and titratable acidity (% lactic acid) were determined based on the procedures described by AOAC [29]. Water holding capacity (WHC) was performed following the procedures described by Ercili-Cura et al. [30]. The WHC was calculated by Equation (2). The LAB viability was determined according to IDF [31] using De Man, Rogosa, and Sharpe (MRS) agar, and colony enumeration was carried out after the anaerobic incubation of the inoculated plates at 37 °C for 72 h.

(2) $$WHC\left(\%\right)=\frac{\mathrm{weight}\mathrm{of}\mathrm{the}\mathrm{pellet}\;(g)}{\mathrm{initial}\mathrm{weight}\;(g)}\times 100$$

### 2.6. Rheological Analyzes

The rheological behavior of yogurts was evaluated following the methodology described by Pacheco et al. [19] using a concentric cylinder rotational rheometer (Brookfield, model R/S plus SST 2000, Toronto, ON, Canada). The data were fitted to the Ostwald-de-Waele model (Equation (3)) using the Curve Expert Professional (version 2.6.5, Hyams Development, Chattanooga, TN, USA) at a 95% confidence level (*p* < 0.05). To calculate the apparent viscosity (η, Pa·s) of the yogurt samples, the values of k and n were used at shear rates of 50 and 100 s^−1^

(3) $$\sigma =k{\dot{\gamma }}^{n}$$

where σ is the stress (Pa), k is the consistency index (Pa·s^n^), γ is the shear rate (s^−1^), and *n* is the flow behavior index (dimensionless).

### 2.7. Optical Microscopy

The microstructural characteristics of the yogurts were evaluated using a binocular optical microscope (Anatomic Opton^®^, Model TIM-18, Campinas, São Paulo, Brazil) coupled with an 8-megapixel portable camera according to the protocol described by Pacheco et al. [19]. A drop of each yogurt sample was deposited on a microscope slide. Objective lens with 10× magnification was used to obtain the images.

### 2.8. Statistical Analysis

The processes were performed in two repetitions and each analysis was carried out in triplicate. The results of particle size, zeta potential, pH, acidity, the viability of lactic acid bacteria, shear rate, flow behavior index, apparent viscosity and WHC were expressed as mean ± standard deviation. Analysis of variance (ANOVA) was performed to evaluate the impact of different treatments and the Tukey test was applied to evaluate significant differences between them (*p* < 0.05). Statistical analyses were performed utilizing Statistica software (version 7.0.61.0, StatiSoft Inc., Tulsa, OK, USA).

## 3. Results and Discussion

### 3.1. Mean Particle Size and Zeta Potential

Table 1 shows mean particle size and zeta potential (ζ) data of goat milk samples supplemented with different concentrations of WPI, pretreated or not by ultrasound-assisted high shear dispersion (US-HSD). The ζ potential is an indicator of the colloidal dispersion stability, and its magnitude represents the level of electrostatic repulsion forces [32]. It was found that supplementing goat milk with WPI provided an increase of up to 47.7% in particle size, with this increase being proportional to the amount of WPI added, which may be correlated with the expressive amount of whey protein in the formulation. In contrast, physical processes (US-HSD) promoted a particle size reduction of up to 42%, with a similarly greater impact on samples without WPI or with 2.5% of whey added.

Such differences can be explained by the effects of US and HSD on milk. US results in turbulence, shear, and the formation of high-pressure zones that are able to reduce the diameter of fat globules, inducing ruptures in casein micelles, and cause the denaturation of whey proteins [13,33,34,35]. The velocity gradient produced by HSD also breaks up milk particles, mainly fat globules [21]. Thus, the association of the two processes maximizes the physical effects on the milk matrix, probably enhancing the quality of the yogurt gel due to greater protein interaction and strong protein network formation [14]. Such effects may enhance the water retention capacity of the gel and the consistency of the yogurt, positively affecting yoghurt quality, which is important from technological and industrial perspectives [13]. Previous results obtained with probe US (ultrasonic probe 20 kHz, 4000 W with a 25% amplitude at 60 °C for 5, 10 and 15 min) showed particle size reduction similar to our results [16]. In this scenario, the solution using bath US + HSD is cost-advantageous compared to probe US [20].

Regarding the zeta potential, it is noted that both the supplementation of goat milk with WPI and the physical processes (US-HSD) increased the electronegativity (*p* < 0.05). For samples not subjected to US + HSD, the reduction in zeta potential (~36%) was achieved with 2.5% WPI supplementation, without a difference in the sample supplemented with 5%. On the other hand, physical processes were able to overcome the impact of supplementation, resulting in zeta potential of approximately—48 mV (~42% greater electronegativity compared with the control sample) for all studied samples. Therefore, the addition of WPI, and mainly the application of physical processes, increased the dispersion of particles by promoting a greater electrostatic repulsion between them, leading to greater physical stability of the milk.

### 3.2. Fermentation Kinetics

**Figure 1:**
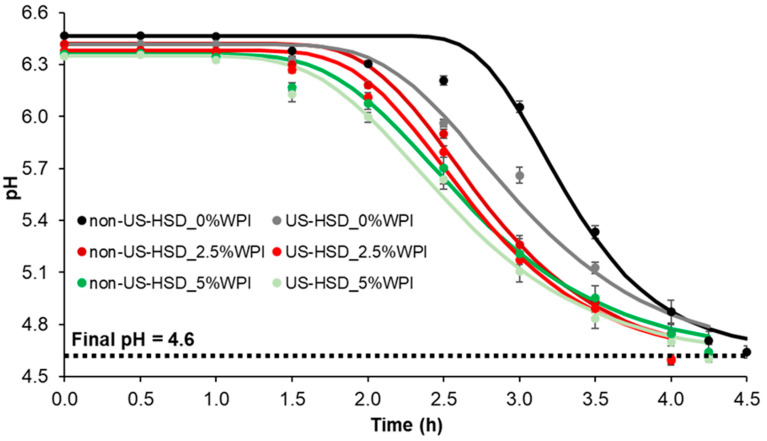
Evolution of pH during yogurt fermentation produced from goat milk pretreated by high shear dispersion assisted by ultrasound-added WPI. US: ultrasound; HSD: high shear dispersion; WPI: whey protein isolate. Dots are experimental data; continuous lines are the predicted data using Equation (1).

At the beginning of fermentation, goat milk showed a pH between 6.34–6.49 and the fermentation process was interrupted when it reached pH 4.6. The pH decline curves (Figure 1) were modeled using Equation (1) (R^2^ = 0.989–0.995), obtaining the values of λ (lag phase time) and μ (maximum rate of pH decline) shown in Table 2.

The control sample exhibited the longest lag phase time and the highest pH decline rate; however, as the percentage of the added WPI increased, the λ and μ parameters decreased (*p* < 0.05) (Table 2). Similar behavior was observed in samples pretreated by US-HSD (*p* < 0.05). This demonstrates that when there was no supplementation of goat milk with WPI and there was no pre-treatment by US-HSD, the microorganisms needed a longer period to uptake or metabolize the nutrients. This can be explained by considering that supplementation increased the availability of nutrients, especially the proteins and peptides important for the symbiotic growth of yogurt cultures [36]. Meanwhile, physical processes may have enhanced the bioavailability of these nutrients through partial protein denaturation and/or particle size reduction [13,33,34,35], facilitating the adaptation of microorganisms to the environment.

On the other hand, the control sample exhibited the most pronounced pH decline after the adaptation step. This can be explained by considering that both WPI supplementation and the application of physical processes may have enhanced the milk buffer capacity [37], requiring greater amount of acid to reach the same pH than the control sample. Finally, from the pH reduction curves during fermentation (Figure 1), it is highlighted that, among the different treatments evaluated, the addition of 2.5% WPI (independent of the US-HSD pre-treatment) reduced the fermentation time in 30 min, compared to the control sample (4 h 30 min). This possibly indicates a positive balance between the increased concentration of growth-promoting factors (due to WPI addition and application of physical processes) and the lower proportion of increased buffering capacity caused by the addition of whey proteins as compared to the highest concentration.

In addition to enhancing productivity and reducing costs, this result can be interesting because a shorter fermentation time can reduce the degree of protein gel network rearrangements, limiting the formation of large pores in the protein network and, consequently, reducing syneresis [13].

### 3.3. pH, Acidity, and LAB Count

After 24 h of refrigerated storage (7 °C), yogurts exhibited pH values ranging from 4.35 to 4.58, with higher values noted for those supplemented with 5% WPI (*p* < 0.05) (Table 3). Additionally, samples presented acidity levels ranging between 0.78–0.89% lactic acid, showing lower acidity values for samples with 5% WPI compared to those with 2.5% WPI (*p* < 0.05) (Table 3).

The LAB counts did not differ among samples, showing values greater than 8 log CFU/mL (*p* > 0.05) (Table 3). This suggests that, despite observed variations in the fermentation process, LAB counts reached similar levels in all samples, regardless of pre-treatment or supplementation. This may be attributed to the longer fermentation time for the non-supplemented or non-physically processed samples, which allowed for similar counts to be achieved at the end of fermentation. Thus, the increase in protein concentration and/or physical processes did not impact the final count of lactic acid bacteria, maintaining them at desirable counts in compliance with regulations, as the counts were higher than the minimum established by the Codex standard for yogurt (≥10^7^ CFU/mL) [38] in all samples.

### 3.4. Rheological Properties

**Figure 2:**
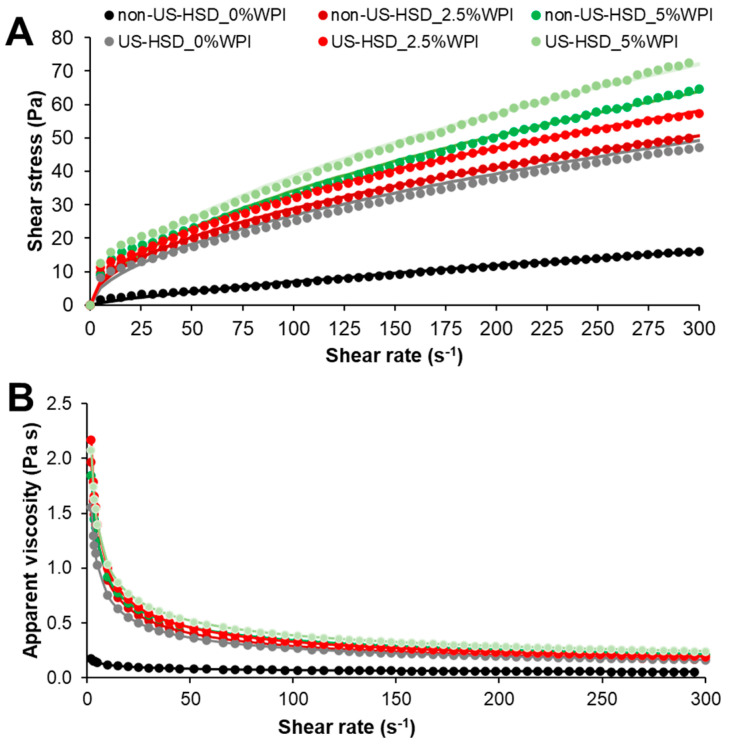
(**A**) Shear stress (σ, Pa) versus shear rate (γ, s^−1^) and (**B**) apparent viscosity (η, Pa s) as a function of shear rate (γ, s^−1^) at 7 °C of yogurt produced with goat milk pretreated by high shear dispersion assisted by ultrasound-added WPI. US: ultrasound; HSD: high shear dispersion; WPI: whey protein isolate.

Figure 2 shows the flow curves that illustrate the correlation between shear stress and shear rate (Figure 2A) and the apparent viscosity and shear rate (Figure 2B) of yogurt samples after 24 h of refrigerated storage.

The curves showed pseudoplastic behavior, in which the shear stress increased non-linearly with the increase in the shear rate, while the apparent viscosity decreased as the shear rate increased, probably due to the breakdown of the aggregates. When all aggregates are dissociated, only colloidal particles remain, and as a result, hydrodynamic forces begin to dominate over other forces, approaching Newtonian behavior, justifying the more consistent viscosity values towards the end [39].

The rheological evaluation data were fitted to Equation (3), and the k and *n* parameters were utilized to calculate the apparent viscosity (η) at two different shear rates: 50 s^−1^ and 100 s^−1^ (Table 4). The experimental data showed an excellent fit to the Oswald-de-Waele model, with R^2^ > 0.99. For the k parameter that represents the consistency index and is related to the protein–protein interactions, it was found that the combination of both strategies did not result in a significant increase when compared to that obtained by isolated strategies. Specifically, supplementation with WPI (independent on the concentration) increased the k value by ~13 times, while the use of physical processes without supplementation led to a 10.6× increase. Therefore, one strategy is sufficient to enhance the consistency of goat yogurt.

Regarding the flow behavior index (*n*), a pseudoplastic behavior (*n* < 1, Table 4) was observed for all samples [40]. The control sample had the highest value for this parameter (0.77) compared to the other samples (*p* < 0.05), indicating that the non-supplemented and non-physically treated sample showed lower resistance to flow. This suggests that this sample is more fluid, and its rheological behavior approaches that of a Newtonian fluid, as also confirmed by the more linear profile in the flow curves (Figure 2).

Conversely, the other samples showed similar flow behavior indexes (*p* > 0.05), ranging from 0.51 to 0.57 (Table 4). Considering that changes in the n parameter indicate alterations in the types of intermolecular forces involved in the gel network [41], these results suggest that both strategies (supplementation or physical processes) can achieve similar responses regarding the consistency and interactions of the gel network.

Table 4 shows the η values in γ of 50 and 100 s^−1^. Based on the obtained results, it was verified that the apparent viscosity greatly increased after 2.5% WPI supplementation and had an additional increase if the supplementation was 5% (*p* < 0.05). In addition, the physical processes also improved the apparent viscosity of the samples (*p* < 0.05) and, contrarily to what has been observed for the other rheological parameters, in this case, the association of the physical process and WPI supplementation had an additive response (*p* < 0.05). Supplementation with 5% WPI increased the apparent viscosity by 5.65 times, and physical processes without supplementation increased by 3.88 times, while the association of these two strategies achieved an increase of 6.41 times (sample pre-treated by US-HSD with 5%WPI) compared to the control sample at a shear rate of 50 s^−1^ (*p* < 0.05). However, it is noted that there was no significant difference in η values between the sample with 5% WPI and the sample pre-treated by US-HSD with 2.5% WPI, thus suggesting that these conditions promoted similar responses regarding the increase in the apparent viscosity of goat yogurt.

The increase in viscosity caused by the increase in the percentage of supplementation can be justified by the higher protein content and, consequently, the total solids of the yogurt produced. Furthermore, this result may also be associated with the effect of heat treatment, which causes denaturation of whey proteins, mainly β-lactoglobulin, enabling their interaction with casein micelles through intermolecular disulfide bonds. Subsequently, these interactions positively influence gel formation, as whey proteins become part of the network formation. As a result, the water retention capacity and viscosity of the yogurt increase [42,43].

Moreover, the cavitation effects generated by US and potentiated by HSD on fat, casein, and whey proteins also contributed to the formation of a stronger network [13,44,45], resulting in a gel with improved rheological properties [12,14] and, consequently, higher apparent viscosity (*p* < 0.05). Thus, for this parameter, an additive effect was observed among the tested strategies, with higher apparent viscosity for the supplemented products whose raw material was pre-processed by US-HSD (*p* < 0.05).

The apparent viscosity results are correlated with the water-holding capacity (WHC) outcomes of the samples (Table 4), where the addition of 5% WPI (US-HSD non-pretreated sample supplemented with 5%) or the application of physical processes (sample pretreated by US-HSD without supplementation—0%) resulted in a WHC increase ranging from 26 to 35% (*p* < 0.05), with no difference between them (*p* > 0.05) (Table 4). In addition, the combination of both strategies (sample pretreated by US-HSD with 5% supplementation) further promoted a significant increase in WHC (48%, *p* < 0.05), suggesting, once again, an additive effect.

### 3.5. Optical Microscopy

**Figure 3:**
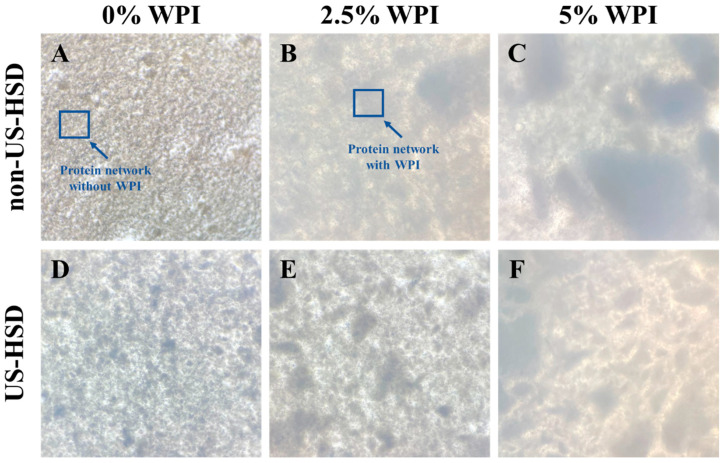
Optical microscopy images of yogurt produced from goat milk not pretreated (**A**–**C**) and pretreated by high shear dispersion assisted by ultrasound (**D**–**F**)-added WPI (**B**,**E**; **C**,**F**) after one day of storage at 7/C. US: ultrasound; HSD: high shear dispersion; WPI: whey protein isolate.

Figure 3 exhibits the optical microscopy images of the yogurt samples after 24 h of refrigerated storage.

The control yogurt showed a poorly crosslinked protein network, demonstrating that goat milk was not capable of forming a yogurt with a strong network, confirmed by the lower values of consistency index, apparent viscosity and WHC shown in Table 4. The poor textural properties of goat yogurt are mainly associated with the reduced α-s_1_-casein content of goat milk compared to other species [46].

In contrast, yogurts with WPI supplementation, as well as those subjected to pre-treatment by US-HSD, showed denser and better-organized protein clusters forming structures containing highly connected networks, indicating direct interactions between casein micelles and denatured whey proteins [6,22,47]. These modifications helped to form a firmer gel with better textural properties, also confirmed by the rheological results discussed in the previous topic.

Based on the obtained results, WPI supplementation demonstrated significant improvements in the apparent viscosity and WHC, which can be advantageous for product quality. However, it is crucial to consider the increase in fixed costs associated with protein addition, especially at the 5% WPI level, despite its positive impact on protein content. On the other hand, the application of the US-HSD process exhibited benefits, including the increased physical stability of the milk (enhanced electronegativity of the zeta potential and reduced particle size), as well as improvements in the rheological and structural properties of the final product. The drawback here lies in the potential initial costs associated with the acquisition and maintenance of the equipment, but with the potential to reduce operational costs in the long run. Finally, the combination of WPI supplementation and physical processes resulted in even more significant enhancements in the apparent viscosity and WHC. Therefore, both strategies offer benefits, and the final decision will depend on the industry’s priorities in terms of costs, product quality, and market positioning. Additionally, considering the importance of sensory quality assessment for overall product acceptance, future studies will be conducted to evaluate sensory attributes under optimized conditions.

## 4. Conclusions

Supplementation with bovine whey protein isolate and the use of combined physical processes (high shear dispersion and ultrasound) as a pre-treatment of goat milk are very promising alternatives to improve the quality attributes of yogurt. Pre-treatment by US-HSD was able to reduce particle size by up to 42%. Both supplementation and physical processes significantly increased the electronegativity of the zeta potential, favoring the physical stability of the milk. The addition of 2.5% WPI was sufficient and adequate to reduce the total yogurt fermentation time by 30 min. Supplementation increased the apparent viscosity and water retention capacity, being proportional to the percentage of WPI added. However, combined use with physical processes promoted greater improvements in these parameters (increases of up to 6.41 times in the apparent viscosity and 48% higher water-holding capacity). Therefore, the decision to use WPI supplementation and apply physical processes (US-HSD), either together or separately, hinges on strategy costs and desired product characteristics.

## Figures and Tables

**Table 1:** Mean particle size and zeta potential of goat milk pretreated by high shear dispersion assisted by ultrasound-added WPI.

Sample		Mean Particle Size (nm)	Zeta Potential (mV)
WPI Supplementation	Pre-Treatment (US-HSD)		
0%	No	978.0 ± 90.1 ^b^	−30.4 ± 5.9 ^c^
2.5%	No	1169.5 ± 135.4 ^b^	−41.6 ± 1.3 ^b^
5%	No	1444.7 ± 119.0 ^a^	−43.4 ± 1.3 ^b^
0%	Yes	593.4 ± 30.5 ^d^	−48.3 ± 0.9 ^a^
2.5%	Yes	679.7 ± 35.9 ^c^	−47.8 ± 1.7 ^a^
5%	Yes	1049.8 ± 156.2 ^b^	−48.8 ± 1.6 ^a^

WPI: whey protein isolated; US: ultrasound; HSD: high shear dispersion. Different superscript lowercase letters (a, b, c, d) (column) indicate significant differences between different treatments by Tukey’s test at 5% probability (*p* > 0.05).

**Table 2:** Fermentation kinetic parameters of goat milk pretreated by high shear dispersion assisted by ultrasound-added WPI.

Sample		λ (h)	μ (h^−1^)	R^2^
WPI Supplementation	Pre-Treatment (US-HSD)			
0%	No	2.73 ± 0.04 ^a^	−1.57 ± 0.06 ^a^	0.991
2.5%	No	2.00 ± 0.03 ^c^	−1.19 ± 0.03 ^b^	0.993
5%	No	1.76 ± 0.11 ^d^	−0.97 ± 0.05 ^c^	0.995
0%	Yes	2.11 ± 0.06 ^b^	−1.03 ± 0.02 ^c^	0.989
2.5%	Yes	1.91 ± 0.05 ^c^	−1.15 ± 0.05 ^b^	0.994
5%	Yes	1.66 ± 0.05 ^d^	−0.98 ± 0.06 ^c^	0.994

λ: lag phase time (h); μ: maximum pH decline rate (h^−1^); US: ultrasound; HSD: high shear dispersion; WPI: whey protein isolate. Significant differences evaluated by the Tukey test (*p* < 0.05) among the samples are indicated by different superscript lowercase letters (a, b, c, d).

**Table 3:** The pH, acidity, and LAB count of yogurt produced with goat milk pretreated by high shear dispersion assisted by ultrasound-added WPI after one day of storage at 7 °C.

Sample		pH	Acidity (% Lactic Acid)	Lactic Acid Bacteria Count (log CFU/mL)
WPI Supplementation	Pre-Treatment (US-HSD)			
0%	No	4.37 ± 0.01 ^b^	0.86 ± 0.06 ^ab^	8.3 ± 0.4 ^a^
2.5%	No	4.35 ± 0.01 ^b^	0.88 ± 0.03 ^a^	8.4 ± 0.4 ^a^
5%	No	4.54 ± 0.04 ^a^	0.80 ± 0.03 ^b^	8.0 ± 0.3 ^a^
0%	Yes	4.38 ± 0.02 ^b^	0.85 ± 0.05 ^ab^	8.0 ± 0.2 ^a^
2.5%	Yes	4.36 ± 0.01 ^b^	0.89 ± 0.02 ^a^	8.5 ± 0.4 ^a^
5%	Yes	4.58 ± 0.02 ^a^	0.78 ± 0.05 ^b^	8.2 ± 0.3 ^a^

Different superscript lowercase letters (a, b) (column) indicate significant differences between the different treatments by Tukey’s test at *p* < 0.05. US: ultrasound; HSD: high shear dispersion; WPI: whey protein isolate.

**Table 4:** Rheological properties and WHC of yogurt produced with goat milk pretreated by high shear dispersion assisted by ultrasound-added WPI.

Sample		Ostwald–De Waele Model			Apparent Viscosity (mPa s)		Water Holding Capacity (WHC) (%)
WPI Supplementation	Pre-Treatment (US-HSD)	k (Pa·s^n^)	*n*	R^2^	γ a 50 (s^−1^)	γ a 100 (s^−1^)	
0%	No	0.20 ± 0.05 ^b^	0.77 ± 0.03 ^a^	0.997	81 ± 9 ^e^	69 ± 6 ^e^	43.0 ± 1.4 ^e^
2.5%	No	2.76 ± 0.35 ^a^	0.51 ± 0.03 ^b^	0.999	405 ± 2 ^c^	288 ± 5 ^c^	51.3 ± 1.2 ^d^
5%	No	2.48 ± 0.35 ^a^	0.57 ± 0.03 ^b^	0.997	458 ± 17 ^b^	340 ± 7 ^b^	57.9 ± 1.7 ^bc^
0%	Yes	2.12 ± 0.80 ^a^	0.55 ± 0.05 ^b^	0.998	315 ± 64 ^d^	233 ± 40 ^d^	54.2 ± 1.5 ^cd^
2.5%	Yes	3.03 ± 0.61 ^a^	0.52 ± 0.05 ^b^	0.998	454 ± 5 ^b^	325 ± 7 ^b^	60.7 ± 2.5 ^ab^
5%	Yes	2.79 ± 0.27 ^a^	0.57 ± 0.01 ^b^	0.996	519 ± 34 ^a^	385 ± 23 ^a^	63.5 ± 1.6 ^a^

Different superscript lowercase letters (a, b, c, d, e) (column) indicate significant differences between different treatments by Tukey’s test at 5% probability (*p* > 0.05). γ: Shear rate; US: ultrasound; HSD: high shear dispersion; WPI: whey protein isolated.

## Data Availability

The original contributions presented in the study are included in the article, further inquiries can be directed to the corresponding author.
